# Alectinib vs. Lorlatinib in the Front-Line Setting for ALK-Rearranged Non-Small-Cell Lung Cancer (NSCLC): A Deep Dive into the Main Differences across ALEX and CROWN Phase 3 Trials

**DOI:** 10.3390/cancers16132457

**Published:** 2024-07-04

**Authors:** Ilaria Attili, Valeria Fuorivia, Gianluca Spitaleri, Carla Corvaja, Pamela Trillo Aliaga, Ester Del Signore, Riccardo Asnaghi, Ambra Carnevale Schianca, Antonio Passaro, Filippo de Marinis

**Affiliations:** 1 Division of Thoracic Oncology, European Institute of Oncology IRCCS, 20141 Milan, Italyantonio.passaro@ieo.it (A.P.);; 2 Division of New Drug Development, European Institute of Oncology IRCCS, 20141 Milan, Italy

**Keywords:** ALK, TKI, brain metastases, cognitive effects, updated results, lipidic profile, survival, intracranial response, toxicity

## Abstract

**Simple Summary:**

This study is an analytic review of the data on the two most used ALK TKIs, alectinib and lorlatinib, used as the first treatment for ALK-positive NSCLC. These drugs have shown promise in separate studies, but there has not been a direct comparison until now. We analyzed data from two major clinical trials that compared these drugs to crizotinib. We found that lorlatinib may be more effective in delaying the progression of the disease compared to alectinib, especially in controlling brain metastases. Although both drugs have side effects, and an alert has been created for the peculiar toxicities of lorlatinib, more people had to stop taking alectinib due to side effects compared to lorlatinib. Overall, the data suggest that lorlatinib might be a better option for patients with this type of lung cancer, but more research is needed to confirm these findings. This information could help clinicians make better treatment decisions for patients with ALK-positive NSCLC.

**Abstract:**

Various next-generation ALK TKIs are available as first-line options for ALK-positive NSCLC, with alectinib and lorlatinib being commonly preferred. However, no direct comparison between them has been conducted, making it impossible to pick a winner. We performed an analytic, ‘non-comparative’ assessment of the two phase 3 pivotal clinical trials showing superiority of alectinib (ALEX) and lorlatinib (CROWN) in comparison to crizotinib. Overall, the two studies were very similar in the study design and patient characteristics, with the exception of the selection and evaluation of brain metastases. PFS hazard ratios numerically favored lorlatinib, both according to the investigator and to BICR. Notably, the 3-year PFS rate was numerically higher with lorlatinib (64%) than with alectinib (46.4%). Despite similar response rates and overall intracranial response, the rate of complete intracranial response was higher with lorlatinib, with a cumulative incidence risk of CNS disease progression at 12 months of 9.4% with alectinib and 2.8% with lorlatinib. The peculiar toxicities of lorlatinib were related to lipidic profile alterations, peripheral oedema and cognitive effects, with no impact on cardiovascular risk nor impairment in quality of life versus crizotinib. Furthermore, the rate of permanent treatment discontinuation due to adverse events was numerically higher with alectinib (26%) than with lorlatinib (7%). In conclusion, despite the immature OS data for both drugs, the efficacy of lorlatinib appears higher than alectinib while maintaining a manageable toxicity profile.

## 1. Introduction

The identification of anaplastic lymphoma kinase (ALK) emerged initially within a subset of anaplastic large-cell lymphomas back in 1994 [1]. It was not until 2007 that the first instance of the echinoderm microtubule-associated protein-like 4 (EML-4)–ALK rearrangement in non-small-cell lung cancer (NSCLC) was documented [2]. ALK gene rearrangements account for approximately 3–7% of all NSCLCs [3,4].

Following the approval of crizotinib as the pioneering ALK tyrosine kinase inhibitor (TKI), there arose a pressing demand for more potent and selective ALK inhibitors to counter resistance [5,6]. Alectinib, a second-generation ALK TKI, exhibited inhibitory activity against several crizotinib or ceritinib-resistant ALK mutations. It notably improved progression-free survival (PFS) and intracranial objective response rate (ORR) in crizotinib-resistant patients compared to platinum-based chemotherapy, with an acceptable safety profile [7]. Subsequently, findings from the global phase 3 ALEX trial showcased the superiority of first-line alectinib over crizotinib, demonstrating enhanced PFS and higher central nervous system (CNS) activity [8,9]. J-ALEX and ALESIA trials confirmed these results in patients from selected countries (Japan, China, South Korea and Thailand) [10,11], and importantly, the 5-year overall survival was 62.5% in the alectinib arm [9]. Similarly, first-line brigatinib, as evidenced by the ALTA 1L study, exhibited improved long-term outcomes compared to crizotinib and emerged as a viable treatment alternative in this context [12]. The advent of the third-generation ALK-TKI lorlatinib, initially designed to combat resistance mechanisms accountable for progression following second-generation TKIs, was a significant milestone [13,14]. The CROWN trial established its efficacy by demonstrating a significant improvement in PFS (HR 0.19) compared to crizotinib [6,15,16]. 

All these ALK-TKI compounds are encountered as possible front-line treatment options in ALK-rearranged NSCLC in the international guidelines [17,18] with higher evaluation (IA; MCBS 4; ESCAT I-A) for alectinib, brigatinib and lorlatinib. However, due to the ‘seniority’ of alectinib approval in this setting (2017), this drug has been the most adopted worldwide, to date. With front-line brigatinib use remaining limited, the benefit observed in the front-line setting with third-generation ALK-TKI lorlatinib appears numerically better than with alectinib or brigatinib and is expected to lead an increasing use of the molecule in the next future. Nevertheless, a direct comparison of the ‘current standard’ alectinib and the new option of lorlatinib has not been conducted, and international debate is ongoing about safety concerns [19,20]. In the current work, we focused on the phase 3 registrational trials of alectinib and lorlatinib (ALEX [8] and CROWN [21] trial, respectively), with the aim of evaluating differences, strengths and limitations in study designs, outcomes and safety, allowing deeper methodological evaluation to inform clinical applicability of both drugs.

## 2. Methods

We performed an analytic, ‘non-comparative’ assessment of the two phase 3 pivotal clinical trials, ALEX [8] and CROWN [21]. For both studies, we conducted a comprehensive search of study protocols and data presented since their first publication on PubMed (https://pubmed.ncbi.nlm.nih.gov/ accessed on 1 April 2024), using the search terms ‘ALEX’, ‘alectinib’, ‘CROWN’, ‘lorlatinib’, ‘update’, ‘survival’, ‘brain’ and ‘patient reported outcomes’. Study protocols were taken from the Supplementary data and Appendix of the original publications. 

Relevant references and cross-citations were also used to identify additional publications through the main international conference proceedings including the American Society of Clinical Oncology, World Conference on Lung cancer, European Lung Cancer Congress and European Society for Medical Oncology (from 2017 to 2023). 

Two researchers (VF and IA) independently screened study protocols and study results to pinpoint the key differences and similarities in the study designs, inclusion and exclusion criteria, endpoints, populations and outcomes with the experimental arms. Discrepancies between the findings by the two researchers were discussed and fully resolved by consensus. Data syntheses were described and presented in analytical tables.

## 3. ALEX vs. CROWN: Main Differences across Phase 3 Studies

### 3.1. Study Designs and Study Populations

In our analytical assessment, we first addressed the evaluation of the two study designs. Both the ALEX and the CROWN were global multicentric phase 3 trials comparing the experimental treatment (alectinib and lorlatinib, respectively) with crizotinib as the standard of treatment in the front-line setting, in a 1:1 randomized assignment [8,21].

Both trials included not previously treated patients with NSCLC harboring ALK translocation detected by Ventana ALK (D5F3) CDx immunohistochemical assay. In the ALEX trial, the positivity must have been confirmed centrally, while the CROWN trial required only that patients must have an archival formalin-fixed, paraffin-embedded (FFPE) tissue specimen available and collected prior to randomization. The stratification factors included both brain metastases (yes vs. no) and race (Asian vs. not Asian), while the ALEX trial included also Eastern Cooperative Oncology Group (ECOG) PS (0–1 vs. 2). The inclusion and exclusion criteria were very similar in the two studies, with an exception related to the inclusion of patients with brain metastases. 

Indeed, the inclusion of patients with untreated baseline brain metastases was allowed in both trials, including leptomeningeal disease, if asymptomatic. However, for patients with neurologically unstable brain metastases, local treatment was required. In the ALEX trial, radiation treatment was required to be completed at least 14 days before enrollment when patients were clinically stable, and corticosteroid administration was allowed with up to 20 mg of prednisone equivalent per day [8]. Conversely, in the CROWN trial, corticosteroid treatment for these metastases was required to have been withdrawn for at least 4 weeks with neurological stability as after local treatment [21].

Also, with comparable timing and methods for radiological response assessments, the evaluation of CNS response differed among the two trials. Indeed, in the CROWN trial, the response of measurable intracranial disease (CNS lesions equal or greater than 5 mm as assessed with MRI) was assessed according to a modified version of the RECIST v1.1 criteria, including up to 5 CNS lesions to be measured in addition to the conventional extracranial evaluation (up to 5 lesions) [21]. Conversely, conventional RECIST v.1.1 criteria were applied in the ALEX trial. Of note, at baseline and at each time re-evaluation point, brain magnetic resonance imaging (MRI) studies were mandatory in both trials [8].

The primary endpoint was progression-free survival in both trials; however, it was differently considered: in the ALEX trial, the endpoint was PFS assessed by an investigator (Inv) [8], whereas in the CROWN trial, it was PFS assessed by blinded independent central review (BICR) [21]. The alternative PFS assessment was the secondary endpoint for each trial. The other secondary endpoints were similar in the two studies.

After evaluating study protocols and methods, we moved to study results and assessed potential key differences in the study populations (Table 1).

The sample size was equally comparable, with 152 patients receiving alectinib and 149 patients receiving lorlatinib in the experimental arms of the ALEX and CROWN trials, respectively [8,21]. The characteristics of patients included in the crizotinib arms were similar in the two trials [8,21].

Demographic characteristics were also similar between the two populations of alectinib and lorlatinib, with a median age of 58 and 61 years, respectively, predominance of the female sex (55% and 56%) and never-smokers (61% and 54%). Baseline disease characteristics, similar for most variables, differed for the proportion of patients with brain metastases: specifically, 42% of patients enrolled in the alectinib arm of the ALEX but only 26% in the lorlatinib arm of the CROWN trial had brain metastases at baseline. Of them, 17% and 6%, respectively, had received prior radiation therapy to the brain (Table 1) [8,21].

### 3.2. Responses and Survival Results

Overall response rates (ORRs) were similar with alectinib (82.9%, 95% CI 76.0–88.5) [8] and lorlatinib (76%, 95% CI 68–83) [21]. The updated ORR is available and confirmed only for lorlatinib (77%, 95% CI 70–84) [15].

At the time of first publication, with similar follow-up of about 18 months (18.6 months in the ALEX and 18.3 months in the CROWN trial), the percentage of patients still on treatment with alectinib and lorlatinib was 55% and 69%, respectively [8,21]. The gap appears even wider with an updated follow-up of about 3 years (37.8 months in the ALEX and 36.7 months in the CROWN trial), with only 34.9% of patients still on alectinib but 61% patients still on lorlatinib (Table 2) [9,15].

The ALEX primary endpoint of PFS assessed by an INV was not reached in the experimental arms in both trials (ALEX: median NE, 95%CI 17.3-NE; CROWN: median NE, 95%CI NE-NE), with PFS rates at 12 months of 68.4% for alectinib and 80% for lorlatinib [8,21].

The CROWN primary endpoint of PFS assessed by BICR was 25.7 months (95% CI 19.9-NE) with alectinib and not reached (95%CI NR-NR) with lorlatinib [8,21]. 

The hazard ratios versus the comparator crizotinib were numerically better in both cases with lorlatinib (PFS by BICR: HR 0.28, 95% CI 0.19–0.41; PFS by INV: HR 0.21, 95% CI 0.14–0.31) as compared to alectinib (PFS by BICR: HR 0.50, 95% CI 0.36–0.70; PFS by INV: HR 0.47, 95% CI 0.34–0.65) [8,21].

At longer follow-up of about 3 years, the PFS results were updated only for the primary endpoint in each trial. In the ALEX trial, the updated PFS assessed by an investigator was 34.8 months (95%CI 17.7-NE) [9], whereas in the CROWN trial, the updated PFS assessed by BICR remained not reached (95%CI NR-NR) [15]. Of note, the 3-year PFS rate was 46.4% with alectinib and 64% with lorlatinib (Table 2), and the updated HR versus crizotinib remained positive for both experimental drugs but was better with lorlatinib (by BICR: HR 0.27, 95% CI 0.18–0.38; by INV: HR 0.19, 95% CI 0.13–0.27) as compared to alectinib (by INV: HR 0.43, 95%CI 68–83) [9,15]. 

Overall survival data are still immature, even at 3-year follow-up, with the median not reached with both alectinib and lorlatinib [9,15].

Of relevance, efficacy data with five-year follow-up have been presented for the CROWN trial, showing that median PFS was still not reached with lorlatinib and there was an updated HR of 0.19 (95% CI 64.3-NR). A 60% PFS rate at 5 years was observed in the lorlatinib arm (versus 8% in the crizotinib arm) [16].

### 3.3. CNS Outcomes

Following the analysis of survival outcomes, we separately evaluated the CNS outcomes observed with the two drugs. 

In the ALEX and CROWN trials, the number of patients with baseline brain metastases were 122 and 78, respectively. Among them, 43 and 30 had brain target lesions. 

Overall, intracranial response rates (IC RR) were similar with alectinib and lorlatinib both among patients (*n* = 21 and *n* = 17) with measurable brain lesions at baseline (IC RR 81% and 82%, respectively) and among patients (*n* = 64 and *n* = 38) with measurable and non-measurable brain lesions at baseline (IC RR 59% and 66%, respectively) (Table 3) [8,21].

Of note, the rate of intracranial complete response among those with measurable brain metastases was 38% with alectinib in the ALEX trial [8,23], whereas it reached 72% with lorlatinib in the CROWN trial [15,21,24]. The median duration of intracranial response was 17.3 months (95% CI, 14.8-NE) and not evaluable (95% CI, NE-NE), respectively (Table 3) [15,21,23,24].

Furthermore, with both drugs, the intracranial objective response rate was higher in those who had not received prior radiotherapy. Indeed, among all patients with baseline brain metastases who previously underwent brain radiotherapy, the IC RR was 36% (95% CI, 57.9–87.0) with alectinib and 25% (95% CI, 56.5–89.7) with lorlatinib. Conversely, among those not receiving radiotherapy, IC RR was 74.4% (95% CI, 18.0–57.5) and 76% (95% CI, 3.2–65.1), respectively (Table 3) [23,24].

The cumulative incidence risk of CNS progression disease at 12 months was 9.4% (HR vs. crizotinib 0.16, 95% CI, 0.10–0.28) with alectinib in the ALEX trial [23] and only 2.8% (HR vs. crizotinib 0.06, 95% CI 0.02–0.18) with lorlatinib in the CROWN trial [21]. In the CROWN trial, the 24-month cumulative incidence risk of CNS progression disease was also calculated and determined to be 5% (HR vs. crizotinib 0.08, 95% CI, 0.04–0.17) [15,24].

At the latest data cutoff, with 3-year follow-up in both studies, the PFS among patients with measurable brain lesions at baseline was 25.4 months (95% CI, 17.3-NE) with alectinib and not reached (95% CI 0.10–0.44) with lorlatinib [9,15].

### 3.4. Safety

#### 3.4.1. Most Frequent Adverse Events 

Both trials used National Cancer Institute Common Toxicity Criteria (NCCN-CTC) version 4: ALEX and CROWN trials utilized v4.0 and 4.3, respectively. Overall, adverse events of any grade during alectinib and lorlatinib treatment occurred in 147 (97%) and 149 (100%) cases, respectively (Table 4) [8,21].

In particular, after a median duration of treatment of 28.1 months, the most frequent adverse events with alectinib were constipation, anemia, fatigue, blood bilirubin increase, peripheral oedema, ALT and AST increase, myalgia, nausea and diarrhea [9].

Conversely, the most common adverse events of any grade reported during lorlatinib treatment were hypercholesterolemia, hypertriglyceridemia, peripheral oedema, weight increase, peripheral neuropathy, cognitive effects, anemia, hypertension, diarrhea, ALT and AST increase [15].

The most frequent adverse events common to both drugs were anemia, increased ALT and AST and peripheral oedema, with similar incidence except for peripheral oedema, reported at higher rates in the lorlatinib group (56%) compared to the alectinib group (19%).

Adverse events leading to dose reduction or temporary discontinuation were reported, respectively, in 20% and 15% of the patients in the alectinib group and 21% and 56% of the patients in the lorlatinib group. However, adverse events leading to permanent treatment discontinuation were reported in 26% of the patients in the alectinib arm and only in 7% of the patients with lorlatinib (Table 4) [8,9,15,21].

Focusing on the peculiar toxicities related to the lipidic profile of patients receiving lorlatinib, the adverse events were manageable (22% required no intervention, 68% required concurrent medication only and 9% required dose interruption or dose reduction and concurrent medication) [15,21].

#### 3.4.2. Grade 3 or Higher Adverse Events

Overall, grade 3 or 4 adverse events (AEs) occurred at a higher frequency in patients treated with lorlatinib (76%) compared to patients who received alectinib (41%) [8,9,15,21].

Among the most common grade 3–4 adverse events, anemia and increased transaminases were more frequent with alectinib (6% and 10%, respectively) [8,9], whereas elevated triglyceride levels, increased weight, elevated cholesterol levels and hypertension were peculiar toxicities of lorlatinib treatment (22%, 20%, 19% and 11%, respectively) [15,21]. Of note, despite the high increase rate of G ≥ 3 in the lipidic profile, a similar incidence of cardiovascular AEs with lorlatinib as compared to crizotinib was observed in the CROWN trial, suggesting the lipidic alteration had no impact on cardiovascular risk [15]. 

The incidence of G ≥ 3 pneumonia was similar (5%) with both drugs (Table 5).

Fatal adverse events reported during alectinib treatment were 5%, but no treatment-related deaths were recorded [8,9].

Conversely, fatal AEs occurred in 3.4% of patients treated with lorlatinib, and two deaths were deemed possibly related to study treatment: one due to acute cardiac failure that occurred approximately 2 months after discontinuation of lorlatinib and the other due to respiratory failure in the setting of infectious pneumonia [15,21].

#### 3.4.3. Focus on Lorlatinib Related CNS Effects

CNS adverse events were distinctive of lorlatinib treatment. Overall, CNS adverse events of any grade occurred in 58 (39%) patients receiving lorlatinib in the CROWN trial [15,24]. Most events were low grade, with only 5% grade 3 and no grade 4 observed. Among all the events observed (*n* = 105), the most frequently reported were cognitive effects (26%), mood effects (17%), speech effects (5%) and psychotic disorders (5%). The majority (59%) required no intervention, and only 2 (1%) required treatment discontinuation (Table 6).

Overall, 56% of CNS events were completely resolved, with over half requiring no intervention. In contrast, most non-resolved events were observed among those managed without any dose modifications [15].

Despite the CNS events, patients in the lorlatinib group had a significantly greater overall improvement from baseline in global quality of life as compared to the standard crizotinib arm, observed as early as cycle 2 and maintained over time [25]. Interestingly, cognitive and mood changes typically present within the first 2 months after lorlatinib administration were managed with dose interruption and reduction without impact in terms of quality of life but only with a not statistically significant trend in cognitive function [25].

### 3.5. Subsequent Treatments

Subsequent treatments were administered in 51 (33%) patients who received alectinib in the ALEX trial and in 33 (22%) patients who received lorlatinib in the CROWN trial. In both trials, the choice of subsequent therapy was at the physician’s discretion and based on treatment availability [9,15].

Of note, a subsequent ALK TKI was given in 39 cases (76%) among patients who received previous alectinib and 21 (64%) among patients who received previous lorlatinib [9,15].

The most frequently received ALK TKIs were crizotinib (28%), lorlatinib (28%), brigatinib (21%) and ceritinib (18%) in patients in the alectinib arm [9]; in the lorlatinib arm, patients mainly received subsequent alectinib (57%), crizotinib (19%), ceritinib (14%) and brigatinib (5%) [15].

The median duration of subsequent ALK TKI treatments was not reported in the ALEX trial and 9.6 months (IQR 2.9–18.1) in the CROWN trial [9,15].

### 3.6. Between-Trial Heterogeneity Assessment of Control Groups

When performing a comparison between two different treatments that were not directly compared with each other but with a third agent as a common comparator, the indirect comparison is also strengthened by the comparison of the outcomes observed with the common comparator. We then evaluated and indirectly compared the outcomes observed in the control arm with crizotinib among the ALEX and CROWN trials [8,21]. 

Baseline characteristics were overall similar in the crizotinib arm of both trials. As already highlighted, the only difference was observed according to the presence or absence of CNS metastases. Consistently with the experimental arm distribution, 38% and 27% of patients who received crizotinib had CNS metastases in the ALEX and CROWN trial, respectively (of them, 17% vs. 7% received previous CNS radiation treatment) [8,21]. 

The responses observed were numerically higher in the ALEX trial than in the CROWN trial among patients in the control arm (ORR 75.5% and 58%, respectively); however, the median duration of response was 11 months in both groups (Table S1). Among patients with CNS metastases, intracranial response rate was similar (26% and 20%, respectively), with a numerically higher rate of complete response observed with crizotinib in the CROWN trial (15%) compared to ALEX (9%) [8,20]. Also, the duration of intracranial response with crizotinib was numerically longer in the CROWN (median 9.4 months) than in the ALEX trial (median 3.7 months) [8,20].

When looking at survival data, crizotinib appeared to have equally performed in the two trials, with a median PFS assessed by the investigator of 11.1 in the ALEX and 9.1 months in the CROWN and PFS determined by BICR of 10.4 and 9.3 months, respectively [8,21].

## 4. Discussion

ALK TKIs have changed the natural history of ALK-positive NSCLC since the ‘crizotinib era’. Alectinib, brigatinib and lorlatinib demonstrated improved responses, CNS outcomes and progression-free survival compared to crizotinib and currently represent alternative front-line treatment options. The data we reviewed on the two phase 3 clinical trials with alectinib and lorlatinib clearly outline a relevant differential magnitude of benefit observed in the CROWN trial, though indirectly compared with ALEX trial, in terms of CNS responses (IC CR 72% vs. 38%), treatment duration (61% vs. 34.9% still on treatment at 3 years) and PFS (3-year PFS rate 64% vs. 46.4%) [9,15], although the differences in inclusion criteria and baseline status may have influenced the CNS results. When looking at front-line brigatinib data, although not the focus of our work, the efficacy results still favor lorlatinib (IC CR 28%, 42% still on treatment at 3 years, 3-year PFS rate 43%) [12]. Interestingly, a subgroup analysis from the CROWN trial showed that the median PFS of lorlatinib in patients harboring variant 3 (a short variant of ALT translocation associated with worse prognosis) was 33 months, while those of patients receiving alectinib and brigatinib were less than 18 months [15,22,26]. 

ALEX, CROWN and ALTA-1L trials were conducted worldwide, with a comparable rate of patients from Asian countries (45%, 44%, 43%, respectively) [8,12,21,27]. Efficacy results with lorlatinib in the Asian subgroup analysis of the CROWN study were consistent with those of the overall population (median PFS not reached, 61% patients still on treatment at 3 years, 73% IC CR) [28]. Conversely, alectinib was specifically assessed in an Asian population in two dedicated phase 3 randomized trials, namely the J-ALEX [10]—evaluating a 300 mg twice daily dose in a Japanese population of untreated and previously chemotherapy treated patients—and ALESIA trials [11]—evaluating a standard alectinib dose in an Asian population in the front-line setting. With follow-up greater than 3 years, the PFS hazard ratio versus crizotinib was 0.37 (95% CI 0.26–0.53) in the front-line population of the J-ALEX trial [29,30] and 0.33 (95% CI 0.23–0.49) in the ALESIA trial [31], which are more similar to that obtained with lorlatinib. However, CNS outcomes, although confirmed positive, remain less marked than those obtained with lorlatinib also in an Asian population (IC CR 35% in the ALESIA trial; 12-month cumulative incidence rate of CNS PD of 5.9% and 7.3% in J-ALEX and ALESIA trials, respectively) [11,32].

In terms of safety, as emerged from metanalyses [33], the spectrum of toxicities of ALK-TKIs differed, with anemia and constipation being more frequent with alectinib, whereas changes in lipid levels were the most frequent AEs associated with lorlatinib. Weight gain and cognitive and mood effects were lorlatinib-specific AEs [33].

The initial alarm for lorlatinib was set on the lipidic profile, including hypercholesterolemia and hypertriglyceridemia, which were observed from the phase 1/2 trial [13,34,35]. However, the alarm was soon clinically lowered, as any grade events were usually asymptomatic and easily managed with appropriate medical therapy (i.e., lipid-lowering agents) and dose interruptions, coupled with dose modifications for more severe (grade ≥ 3) AEs [36]. In addition, hypercholesterolemia and hypertriglyceridemia were not common reasons for dose delay (3.4% and 4.7%, respectively) or dose reduction (0.7% and 1.7%, respectively) and did not result in any permanent discontinuations across the phase 1/2 study [34]. Importantly, a post hoc analysis from the CROWN trial has shown that the high incidence of dyslipidemia did not translate to a higher risk of cardiovascular events [15].

Indeed, although the rate of temporary discontinuation due to AEs with lorlatinib was high (56%), the rate of permanent drug discontinuation was very low with lorlatinib compared to alectinib (7% vs. 15%, respectively). The low discontinuation rate with lorlatinib is confirmed from the phase 2 study (3%) [35]. For thoroughness, the rate of AEs leading to treatment discontinuation with brigatinib in the ALTA-1L trial was 13% (with 72% dose interruption and 44% dose reduction) [12].

The major point of attention for lorlatinib remains focused on the neurocognitive adverse events. 

Actually, psychiatric symptoms were observed across ALK TKIs. Indeed, in a large retrospective analysis of spontaneous reports submitted to the Food and Drug Administration Adverse Events Reporting System, psychiatric reports emerged for crizotinib, lorlatinib, alectinib, ceritinib and brigatinib (0.3%, 2.8%, 0.7%, 0.6% and 1.2% of all AEs suspected to be related to the specific compound, respectively) [37]. 

The mechanism underlying psychiatric adverse events (AEs) caused by ALK inhibitors remains unclear, despite ALK’s involvement in regulating the dopamine D2 receptor (D2R), which is implicated in psychiatric disorders [38]. Prolonged exposure to dopamine leads to D2R desensitization, with ALK inhibition hindering dopamine-mediated inhibition in mice models. Additionally, ALK promotes D2R desensitization by facilitating endocytosis in response to prolonged dopamine stimulation [38,39].

The increased occurrence of psychiatric AEs with lorlatinib may be due to its ability to penetrate the blood–brain barrier (BBB) and low propensity for P-glycoprotein (P-gp) efflux, resulting in its accumulation in the CNS [40]. This accumulation, attributed to lorlatinib’s macrocyclic structural characteristics, may exacerbate psychiatric AEs [41]. Furthermore, a speculative hypothesis is that lorlatinib might act as a catalyst following prolonged D2R desensitization induced by earlier-generation ALK TKIs in pretreated patients.

Of note, brain metastases, brain radiation, psychiatric illness, psychiatric medications, antiepileptics and stimulants were associated with developing cognitive effects in a retrospective study of patients receiving lorlatinib in two prospective trials [42]. In the same study, psychiatric illness, psychiatric medications, benzodiazepines and brain surgery were associated with developing mood effects, whereas brain radiation and antiepileptics were associated with speech effects [42].

Consistent with these findings, 71.8% of 117 patients with CNS effects in the lorlatinib phase 1/2 trial had baseline brain metastases, and 26.2% of patients with CNS effects and baseline CNS metastases had received prior whole-brain radiation therapy [34]. However, as occurred in the CROWN study, CNS adverse effects were generally mild in severity and improved or resolved upon dose modifications (grade 3 mood disorders 1.4%, grade 3 cognitive disorders 1.7%, no grade 4 events) [13,24,34,35].

## 5. Conclusions

In conclusion, CNS disorders are adverse events to ALK TKIs, more frequent with lorlatinib. Therefore, it is crucial for medical oncologists, patients and caregivers to remain vigilant regarding any changes in cognition, mood or speech, prompting timely specialist evaluation or adjustments in dosage [36]. Despite the absence of direct comparative studies, the accumulating evidence overwhelmingly supports the front-line use of lorlatinib, poised to emerge as the new standard treatment, with anticipation of future head-to-head trials stemming from academic endeavors.

## Figures and Tables

**Table 1:** Summary of main baseline characteristics across trials.

	ALEX Trial (Alectinib Arm)	CROWN Trial (Lorlatinib Arm)
**Age**		
Mean, no. ± SD	56.3 ± 12.0	59.1 ± 13.1
Median, no.	58.0	61
Range	25–88	51–69
**Sex**		
Male, no. (%)	68 (45)	65 (44)
Female, no. (%)	84 (55)	84 (56)
**Smoking status**		
Never smoked, no. (%)	92 (61)	81 (54)
Previous smoker, no. (%)	48 (32)	55 (37)
Current smoker, no. (%)	12 (8)	13 (9)
**Current stage of disease**		
IIIA, no. (%)	0	1 (1)
IIIB, no. (%)	4 (3)	13 (9) ^1^
IV, no. (%)	148 (97)	135 (91)
**CNS metastases, no. (%)**		
Yes	64 (42)	38 (26)
No	88 (58)	111 (74)
**Previous brain radiation, no. (%)**		
Yes	26 (17)	9 (6)
No	126 (83)	140 (94)

^1^ One patient in CROWN trial was assessed according to 8th AJCC edition instead of 7th; SD, standard deviation; CNS, central nervous system.

**Table 2:** Comparison of main results across trials.

	First Publication *		3-Year Results *	
	Alectinib	Lorlatinib	Alectinib	Lorlatinib
Intention to treat population, *n*	152	149	152	149
Median follow-up, months (95% CI)	18.6 (0.5–29)	18.3 (NA)	37.8 (0.5–50.7)	36.7 (31.3–41.9)
Median duration treatment, months (95% CI)	17.9 (0–29)	NA (NA)	28.1 (NA)	33.3 (13.9–39.8)
Patients still on treatment at data cutoff, %	55%	69%	34.9%	61%
ORR, %	82.9%	76%	82.9 §	77%
Median duration of response, months (95% CI)	NE (NE-NE)	NE (NE-NE)	33.1 mos §	NR (NR-NR)
PFS by INV				
Median, months (95% CI)	NE (17.7-NE)	NR (NR-NR)	34.8 (17.7-NE)	NR (NR-NR)
PFS rate	68.4% at 12 months	80% at 12 months	46.4% at 3 years	-
HR vs. crizotinib (95% CI)	0.47 (0.34–0.65)	0.21 (0.14–0.31)	0.43 (0.32–0.58)	0.19 (0.13–0.27)
PFS by BICR				
Median, months (95% CI)	25.7 (19.9-NE)	NR (NR-NR)	Not repeated	NR (NR-NR)
PFS rate	-	78% at 12 months	-	64% at 3 years
HR vs. crizotinib (95% CI)	0.5 (0.36–0.70)	0.28 (0.19–0.41)	-	0.27 (0.18–0.39)
OS				
Median, months (95% CI)	NE	NE	NR	NE
OS rate	-	-	62.5% at 5 years	-
HR vs. crizotinib (95% CI)	0.76 (0.48–1.20)	0.72 (0.41–1.25)	0.67 (0.46–0.98)	-

* Results with crizotinib were similar in both trials—not reported; § data extracted from the publication at median follow-up of 27.8 months [22]; BICR, blind independent central review; CI, confidence interval; CNS, central nervous system; HR, hazard ratio; NA, not available; NE, not estimated; NR, not reached; PFS, progression-free survival; ORR, overall response rate; OS, overall survival; PD, progression disease.

**Table 3:** Comparison of main CNS results across trials.

	First Data Cutoff *			Latest Data Cutoff *	
	Alectinib	Lorlatinib		Alectinib	Lorlatinib
Intention to treat population, *n*	152	149		152	149
Median follow-up, months	18.6 (0.5–29)	18.3 (NA)		37.8 (0.5–50.7)	36.7 (31.3–41.9)
**CNS outcomes**					
12 m cumulative incidence rate of CNS PD, %	9.4%	2.8%	24 m cumulative incidence rate of CNS PD, %	-	5%
CS HR vs. crizotinib	0.16 (0.10–0.28)	0.06 (0.02–0.18)		-	0.08 (0.04–0.17)
Patients with measurable CNS lesions at baseline, *n*	21	17		-	18
IC-ORR, %	81%	82%		-	83%
Complete response, %	38%	71%		-	72%
Median IC-DoR, months	17.3 (14.8-NE)	NE (NE-NE)		-	NR (NR-NR)
Patients with measurable and non-measurable CNS lesions at baseline, *n*	64	38		-	37
ORR, %	59%	66%		-	65%
Complete response, %	45%	61%		-	59%
Median duration of response, months	NE (17.3-NE)	NE (NE-NE)		-	NR (NR-NR)

* Results with crizotinib were similar in both trials—not reported; CNS, central nervous system; CS HR, cause-specific hazard ratio; M, months; NE, not estimated; NR, not reached; ORR, overall response rate; PD, progression disease; IC, intracranial; IC-DoR, IC duration of response.

**Table 4:** Most frequent AEs across trials.

	Alectinib	Lorlatinib
Any grade, *n* (%)	147 (97)	149 (100)
events, *n* (%)	Constipation, 56 (36)	Hypercholesterolemia, 108 (72)
	Anemia, 40 (26)	Hypertriglyceridemia, 99 (66)
	Fatigue, 34 (22)	Peripheral oedema, 83 (56)
	Blood bilirubin increased, 33 (22)	Weight increased, 65 (44)
	Peripheral oedema, 29 (19)	Peripheral neuropathy, 60 (40)
	ALT increased, 27 (18)	Cognitive effects, 38 (26)
	Myalgia, 26 (17)	Anemia, 33 (22)
	AST increased, 26 (17)	Hypertension, 33 (22)
	Nausea, 25 (16)	Diarrhea, 33 (22)
	Diarrhea, 24 (16)	ALT increased, 27 (18)
	Upper respiratory tract infections, 21 (14)	Mood effects, 26 (17)
Dose reduction *n*, (%)	31 (20)	32 (21)
Temporary discontinuation *n*, (%)	40 (26)	84 (56)
Permanent discontinuation *n*, (%)	22 (15)	11 (7)

**Table 5:** Grade 3 or higher adverse events (AEs) across trials.

	Alectinib	Lorlatinib
Grade ≥ 3, *n* (%)	79 (52)	113 (76)
Events, *n* (%)	Anemia, 9 (6)	Hypertriglyceridemia, 34 (22)
	AST increased, 8 (5)	Weight increased, 30 (20)
	ALT increased, 7 (5)	Hypertriglyceridemia, 29 (19)
	Pneumoniae, 7 (5)	Hypertension, 17 (11)
	Urinary tract infection, 6 (4)	Pneumoniae, 7 (5)
	Hypokalemia, 4 (3)	Oedema, 6 (4)
	Blood bilirubin increased, 4 (3)	ALT increased, 4 (3)
	Acute kidney injury, 4 (3)	Dyspnea, 4 (3)
	Back pain, 3 (2)	Respiratory failure, 4 (3)

**Table 6:** CNS effects with lorlatinib.

Grade		Any	G1	G2	G3	G4
Any CNS AE		58 (39)	34 (23)	16 (11)	8 (5)	0 (0)
Cognitive effects		38 (26)	23 (15)	10 (7)	5 (3)	0 (0)
Mood effects		26 (17)	14 (9)	10 (7)	2 (1)	0 (0)
Speech		8 (5)	6 (4)	1 (1)	1 (1)	0 (0)
Psychotic effects		7 (5)	5 (3)	1 (1)	1 (1)	0 (0)
Interventions in CNS events	*n*, (%)	AE Outcome				
	103 *	Completely resolved	Partially resolved		Not resolved	
No intervention	61 (59)	32 (31)	1 (1)		28 (27)	
Concomitant medication only	14 (14)	8 (8)	0 (0)		6 (6)	
Dose reduction only	4 (4)	3 (3)	0 (0)		1 (1)	
Dose interruption only	15 (15)	12 (12)	2 (2)		1 (1)	
Combined intervention	7 (7)	3 (3)	1 (1)		3 (3)	
Permanent discontinuation	0 (0)	0 (0)	0 (0)		0 (0)	

* Two events excluded from the analysis.

## Data Availability

No new data were created or analyzed in this study. Data sharing is not applicable to this article.

## Supplementary Materials

The following supporting information can be downloaded at: https://www.mdpi.com/article/10.3390/cancers16132457/s1, Table S1: Main efficacy results in the control arm with crizotinib in the Alex and Crow trials.
