# Supplementary material for: Alectinib vs. Lorlatinib in the Front-Line Setting for ALK-Rearranged Non-Small-Cell Lung Cancer (NSCLC): A Deep Dive into the Main Differences across ALEX and CROWN Phase 3 Trials
Source: Cancers (Basel). 2024 Jul 4;16(13):2457. doi: 10.3390/cancers16132457 (PMC11240527; doi:10.3390/cancers16132457)
Supplement: Supplementary file 1 [file cancers-16-02457-s001.zip › cancers-3083020-supplementary.pdf]

Table S1. Main efficacy results in the control arm with crizotinib in the Alex and Crow trials.

	**Alex Trial (Crizotinib arm)**	**Crown Trial (Crizotinib arm)**
**Intention to treat population, n**	151	147
**Median follow-up, months (95% CI)**	17.6 (0.3 -27)	14.8 (NA)
**Patients still on treatment at data cutoff, %**	30%	22%
**ORR, %**	75.5%	58%
**Median duration of response, months (95% CI)**	11.1 (7.9 - 13)	11.0 (9 -12.9)
**PFS by INV**		
**Median, months (95% CI)**	11.1 (9.1 – 13.1)	9.1 (7.4 - 10.9)
**PFS rate**	48.7% at 12 months	35% at 12 months
**PFS by BICR**		
**Median, months (95% CI)**	10.4 (7.7-14.6)	9.3 (7.6 – 11.1)
**PFS rate**	-	39% at 12 months

BICR, blind independent central review; CI confidence interval; PFS, progression free survival; ORR, overall response rate
